# A case report of community-acquired *Raoultella ornithinolytica* infection in a healthy, young individual

**DOI:** 10.1186/s12879-021-06799-w

**Published:** 2021-10-24

**Authors:** Xiaonan Chen, Xinjian Zhou, Jun Cao, Ke Ma, Zhijie Xia

**Affiliations:** Emergency and Acute Critical Care Department, Huashan Hospital North, Fudan University, Shanghai, China

**Keywords:** *Raoultella ornithinolytica*, Community-acquired infection, Anti-allergic treatment

## Abstract

**Background:**

*Raoultella ornithinolytica* is a Gram-negative bacillus that resembles Klebsiella. This bacterium is present in many soil and aquatic environments and is a major causative agent of healthcare-associated infections (HAIs) in medical staff. Clinically, it has been reported to contribute to nosocomial infections in patients that include but are not limited to gastrointestinal, skin, and genitourinary tract infections. These complications are most common in hospitalized patients with underlying immunodeficiency, multiple comorbidities, or those receiving invasive surgery.

**Case presentation:**

We present a case of a 25-year-old patient with a *R. ornithinolytica* infection. The patient had no history of any disease. Her main complaints were high fever, a scattered maculopapular rash, and superficial lymph node enlargement (SLNE). Peripheral blood samples were collected for high-throughput sequencing analysis to identify pathogenic microorganisms. The results confirmed a *R. ornithinolytica* infection, which was treated successfully using meropenem. Loratadine was also administered to treat the patient’s compromised skin condition caused by an allergic reaction.

**Conclusions:**

To our knowledge, this is the first case of a systemic maculopapular rash and superficial lymphadenopathy caused by a *R. ornithinolytica* infection acquired at the community level. Based on this case, we recommend a combination of antibiotic and antiallergic drugs to treat a *R. ornithinolytica* infection and associated allergic reaction to the bacteria.

## Background

**Fig. 1:**
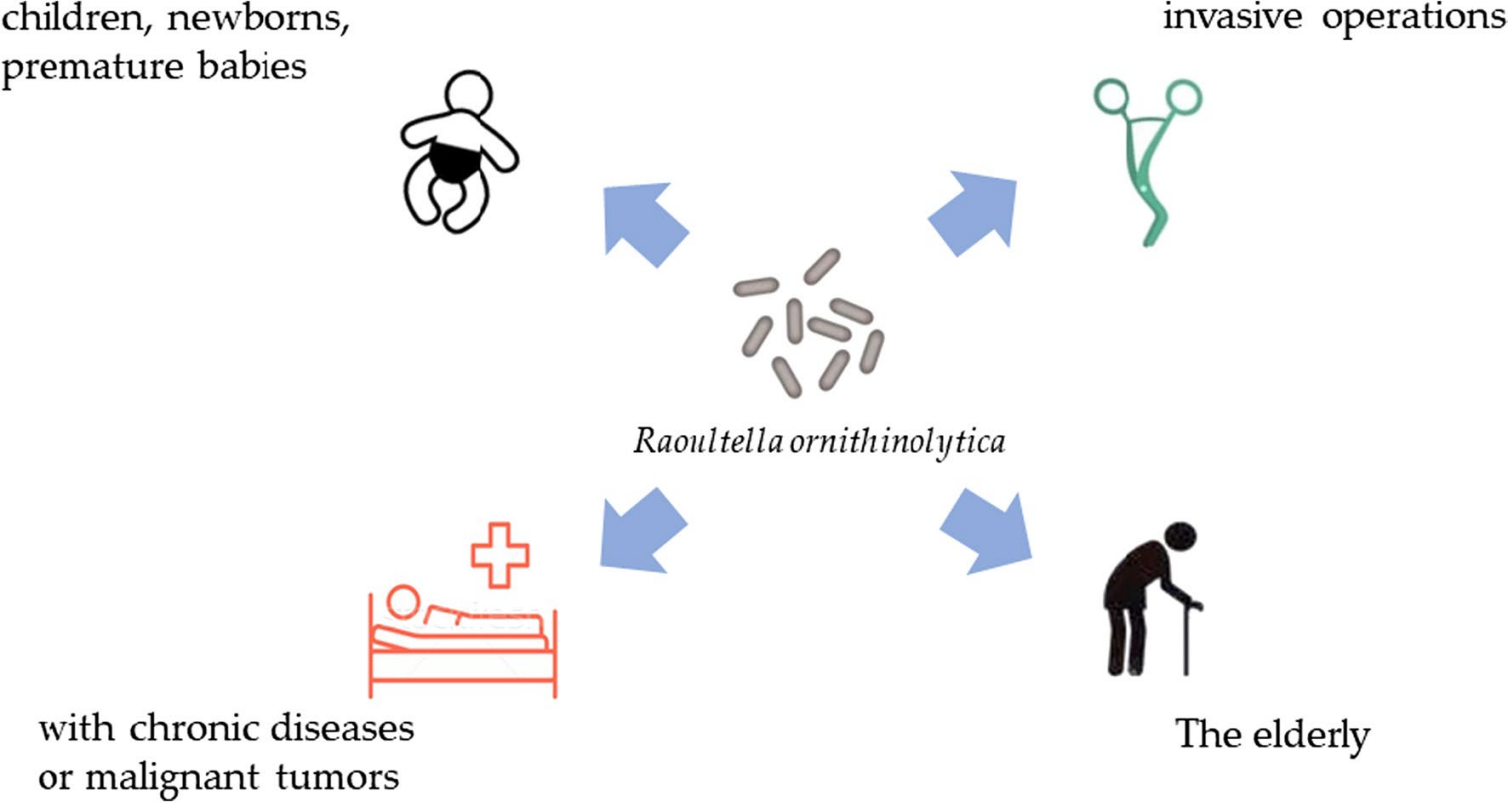
The risk factors of the *Raoultella ornithinolytica* infection and the susceptible population

*Raoultella ornithinolytica* is a Gram-negative enterobacteria that is encased within a capsule. The bacterium is immobile and is a facultative anaerobe. The bacterium was initially named *Klebsiella ornithinolyticus* in 1989, however in 2001 it was included in the genus *Raoultella* together with *Klebsiella* lyorniferum, plant *Klebsiella*, and native *Klebsiella*. This recategorization was based on the finding that its *16Sr-RNA* and *rpoB* genes were inconsistent with those of *Klebsiella* [1]. *R. ornithinolytica* can be isolated from water, soil, plants, and occasionally from mammalian mucosa, including human mucosal specimens. For individuals (Fig. 1) with low immunity, such as the elderly and those with underlying chronic diseases and malignant tumors, secondary infections with *R. ornithinolytica* are common during hospitalization or following invasive operations. The bacterium can also cause fatal infections in children [5], newborns, and especially premature babies [2]. In recent years, several strains of *R. ornithinolytica* resistant to multiple antibiotics have been reported [3, 4], which has resulted in an increased number of HAIs in control departments. To our knowledge, *R. ornithinolytica* infections have been reported only rarely in healthy people in the community. This is the first reported case of a systemic maculopapular rash and superficial lymphadenopathy caused by *R. ornithinolytica* infection acquired at the community level. Based on this case, we innovatively recommend a combination of an antibiotic and antiallergic drug to treat the *R. ornithinolytica* infection and the resulting allergic reaction to the bacteria.

## Case presentation

**Fig. 2:**
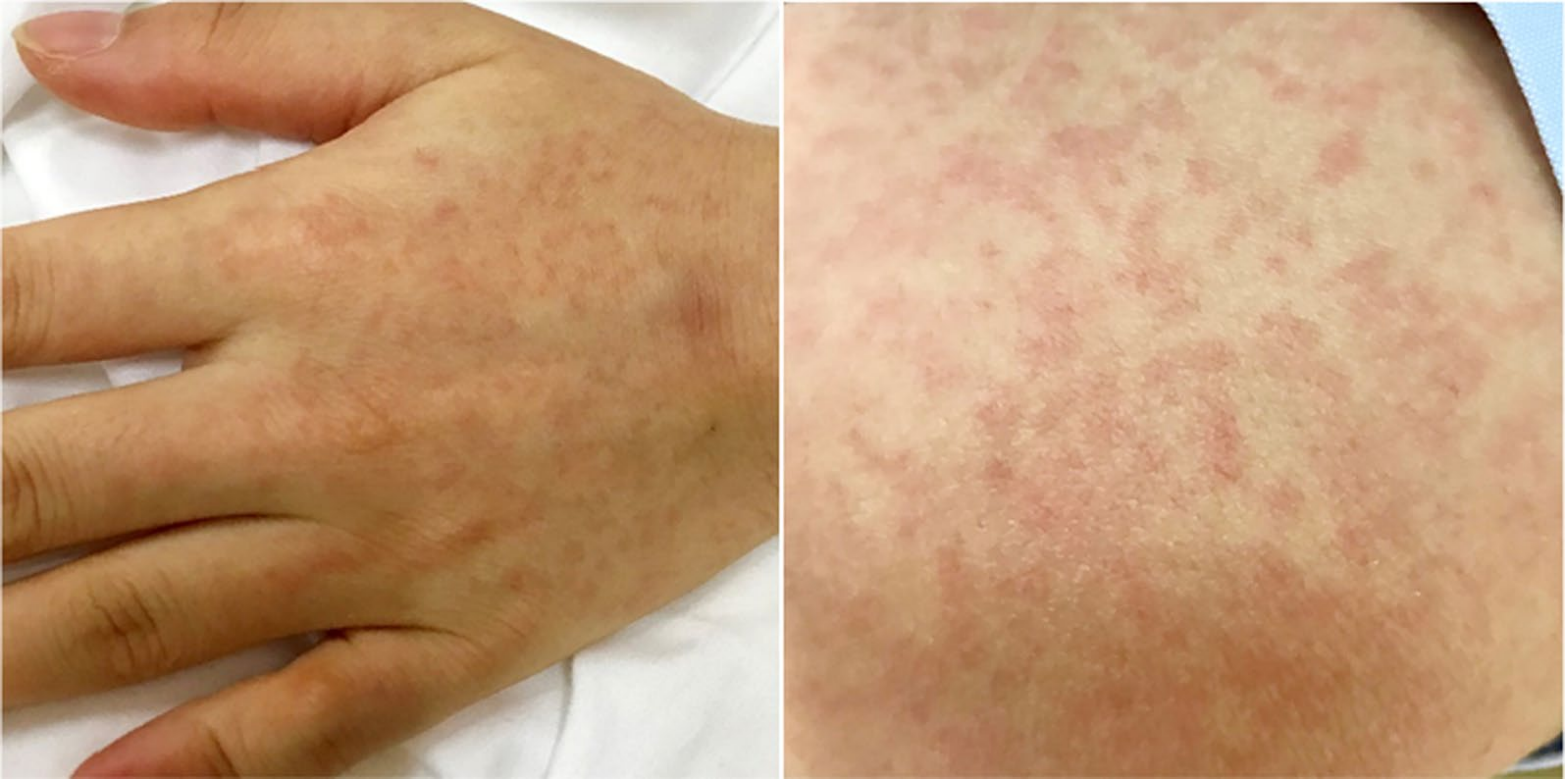
Scattered maculopapular rash on the patient’s skin

**Fig. 3:**
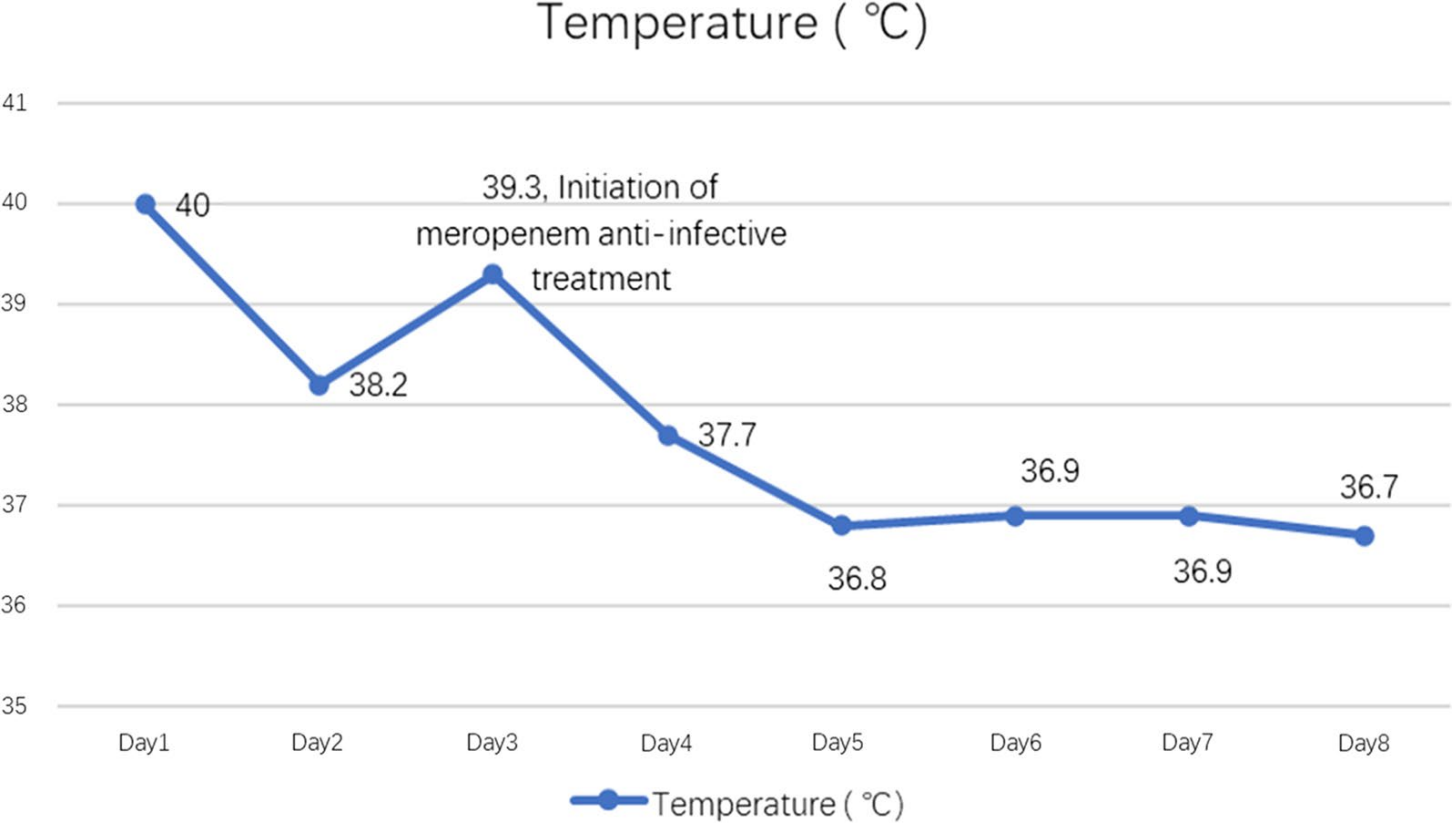
Changes in the patient’s body temperature after admission

A 25-year-old female patient with no significant past medical or surgical history was admitted to the emergency room due to fever, chills, paroxysmal cough, and scattered rashes (Fig. 2). Before the onset of illness, the patient was a general staff member of a company and had been involved in picking strawberries at a suburban farm. The results of blood tests at admission were an increased WBC count of 11.93 × 10^9^/L, an increased proportion of neutrophils of 85%, and a raised CRP level of 24.62 mg/L. The patient failed to respond to oral cefixime treatment and her body temperature progressively increased to 40 °C (Fig. 3). The patient’s vital signs were a blood pressure of 116/70 mmHg, heartrate of 86 bpm, SpO_2_ of 100%, and respiratory rate of 19 beats/min. Physical examination revealed that the patient’s bilateral neck, submandibular, and left groin regions had multiple enlarged lymph nodes, some of which were tender and others slightly hardened. The pharynx was congested and the patient’s skin was red with scattered itchy maculopapular rashes.

**Fig. 4:**
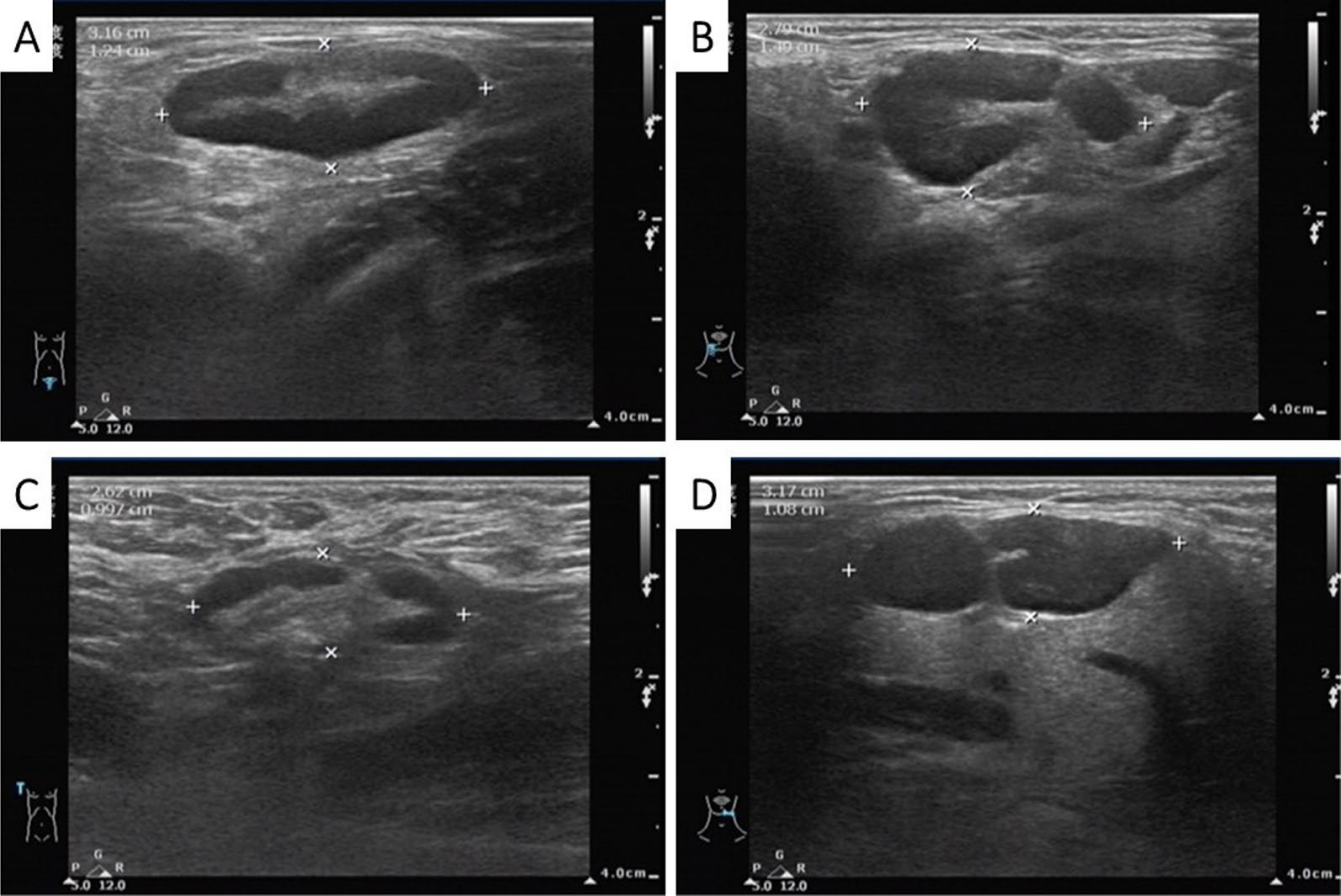
The enlargement of the superficial lymph nodes under B-ultrasonic examination. (**A**: inguinal lymph node 3.16 cm × 1.24 cm; **B**: submandibular lymph node 2.79 cm × 1.49 cm, **C**: supraclavicular lymph node 2.62 cm × 0.997 cm, **D**: Neck lymph node 3.17 cm × 1.08 cm)

**Fig. 5:**
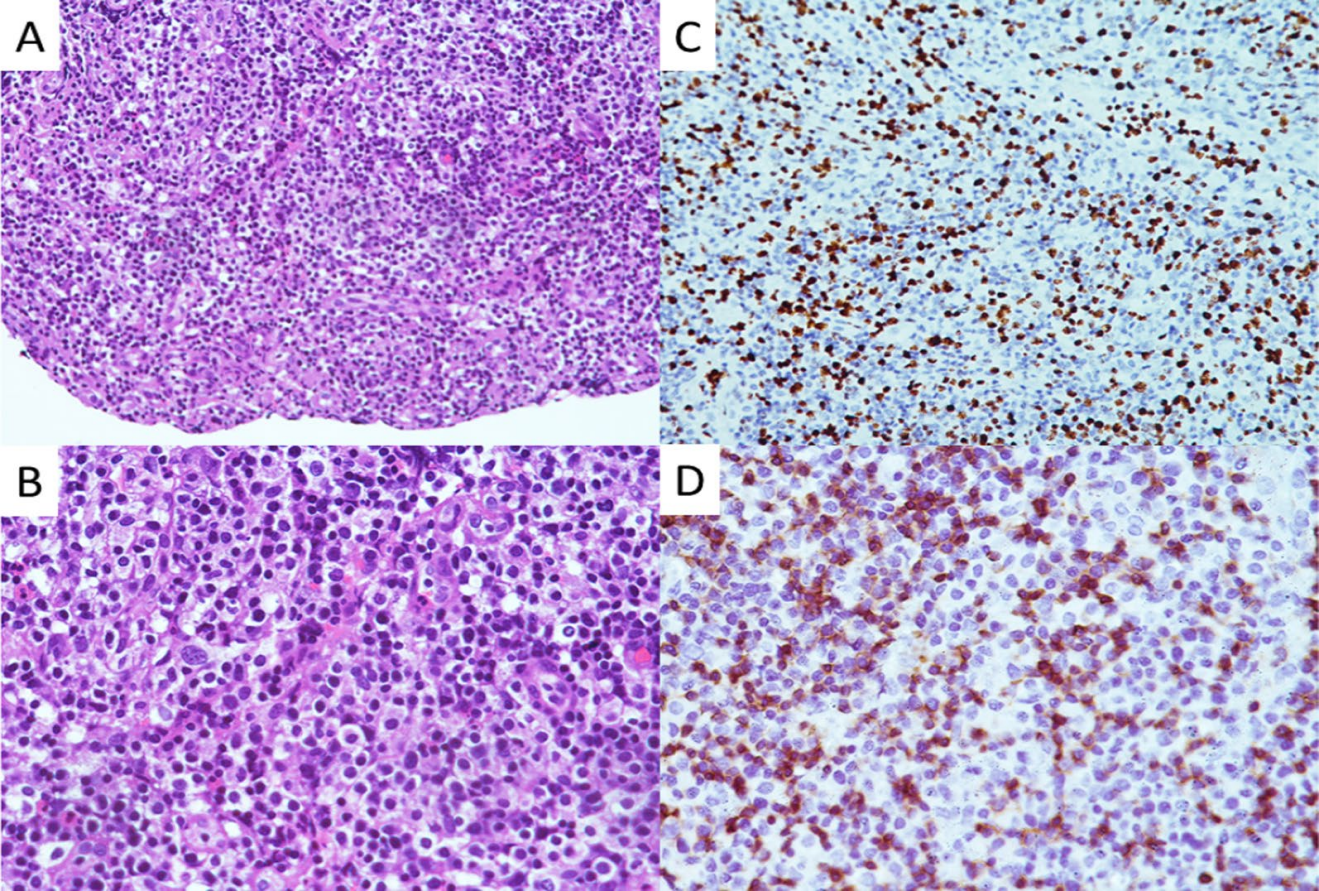
The photography of the left inguinal lymph node pathology. (**A**, **B**: HE staining of the left inguinal lymph node; **C**, **D**: Immunohistochemical staining of the left inguinal lymph node)

**Fig. 6:**
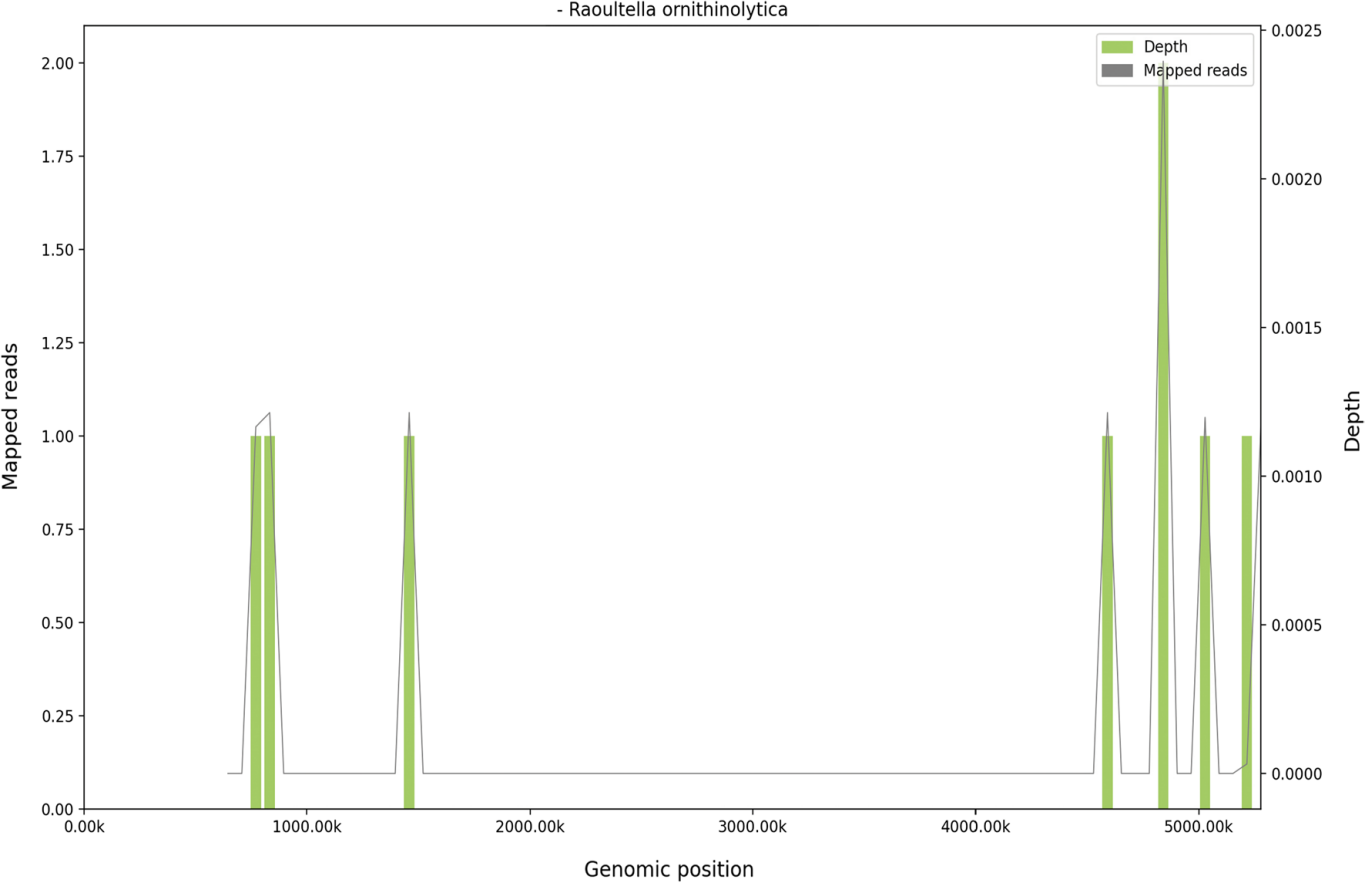
The complete genome coverage map of *Raoultella ornithinolytica* strain

The results of blood tests performed after admission are shown in Table 1. B-ultrasonic examination (Fig. 4) showed that the lymph nodes of the bilateral submandibular glands, bilateral neck, bilateral supraclavicular, bilateral inguinal were swollen. An abdominal CT also revealed enlarged lymph nodes in the left groin. During hospitalization, the drug regime was adjusted several times (amoxicillin and potassium clavulanate, moxifloxacin, and ganciclovir), although the patient’s response to these drugs was not satisfactory. Because the cause of the illness remained unknown we performed additional tests that included IgM antibody examination for common pathogens using a broad range of autoantibodies and blood and sputum cultures. None of these tests were positive for bacteria or fungi. The nucleic acid test for COVID-19 was negative. A pathological biopsy specimen of a left inguinal lymph node was performed with histology showing structural disorder and cortical atrophy of the node and T cell proliferation in the paracortical area with infiltration of a large number of plasma cells (Fig. 5). The patient was diagnosed histologically with reactive hyperplasia. In order to further investigate possible causes for the infection, peripheral blood samples were collected on the 3rd day of admission for next-generation sequencing analysis to detect pathogenic microorganisms. The results showed that *R. ornithinolytica* was present, although no drug resistance genes were detected in the analysis (Fig. 6).

**Table 1:** Changes in infection index levels after admission

	WBC (× 10^9^/L)	NEUT %	CRP (mg/L)	PCT (ng/mL)
Day1	11.93	85.8	24.62	–
Day2	10.79	86.7	166.19	0.84
Day3	8.76	90.8	133.71	0.77
Day4	6.51	46.2	15.71	0.17

Limits values: WBC (4.0–10.0) × 10^9^/L; NEUT 50–70%; CRP 0–5 mg/L; PCT < 0.5 ng/mL

**Fig. 7:**
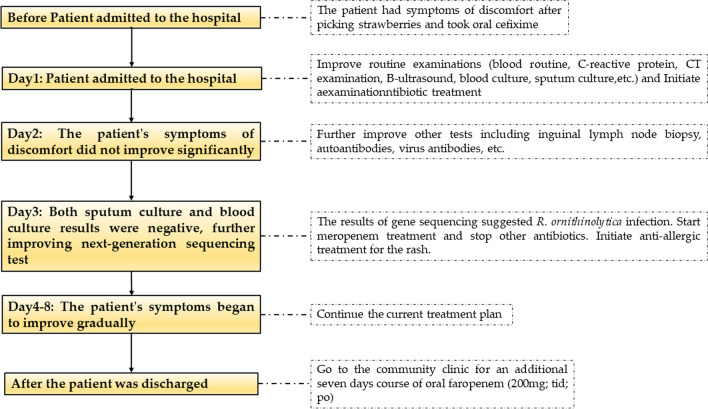
The timeline of the entire course of diagnosis and treatment

The treatment regimen was adjusted in accordance with the sequencing results. Meropenem (1 g; q8h; ivgtt) in combination with loratadine (10 mg; qn; po) were administered for 6 days, with the patient’s body temperature decreasing progressively to normal levels on the 2nd day of treatment (day 4). In addition, the rash gradually disappeared and the inflammatory reactions gradually decreased as shown by the results of the blood tests summarized in Table 1. On the 4th day of the final treatment it was observed that the size and tenderness of the enlarged lymph nodes had also decreased. Seven days after the final adjusted treatment, the patient gradually recovered and was discharged from the central hospital to the community clinic for an additional 7 days course of oral faropenem (200 mg; tid; po). The timeline of the entire course of diagnosis and treatment is shown in Fig. 7.

## Discussion and conclusions

*Raoultella ornithinolytica* infection is a new type of infectious disease that has emerged globally, with the overall incidence currently showing an increasing trend. *R. ornithinolytica* is a common respiratory, urinary, and bloodstream infection that follows catheter placement and artificial joint replacement [5–8]. This occurs because the bacteria form a biofilm on the inner surface of artificial implants such as catheters leading to HAIs [9]. In some cases, appendicitis, liver abscess, bacteremia and septic shock have been reported [10, 11] with some infections also causing oral ulcers and facial nerve palsy [12]. In the present case, the patient had abnormally enlarged superficial lymph nodes. These clinical manifestations associated with *R. ornithinolytica* infection have not been reported previously. The occurrence of similar cases should therefore be monitored closely to provide sufficient data to better understand the clinical features and risk factors of this type of infection.

In many of the cases reported to date, *R. ornithinolytica* has been considered as an opportunistic pathogen that rarely infected humans. These infections are most prevalent in individuals with impaired immune function. In the present case, the patient had no history of abnormal immune function and we infer that her disease was due to an opportunistic infection caused by contact with soil or plants whilst picking strawberries. In previous case reports [1, 3, 5, 8, 9, 11], MALDI-TOF MS technology was used to identify the *R. ornithinolytica* infections. However, in our case, due to cost constraints, the clinical laboratory of our hospital is not equipped with the equipment needed to carry out MALDI-TOF MS analyses. Blood culture has several disadvantages as it requires a large volume of blood (20–30 ml), takes a long time to obtain results, is susceptible to contamination, and prone to false negative results due to culture conditions. We therefore finally used next generation sequencing analysis to identify the pathogenic microorganisms in the surrounding blood and then compared them with the pathogenic microorganism database to detect possible pathogenic sources. This technique only requires 5–8 mL of blood samples and we consider that it has the potential to rapidly identify clinical pathogens.

*Raoultella ornithinolytica* has been shown to express β-lactamase and is therefore not sensitive to clinical routine antibiotic intervention [13]. Case reports in retrospective studies have shown that 80% of *R. ornithinolytica* strains are resistant to amoxicillin, 17% to amoxicillin-carat tretinoin, 15% to piperacillin/tazobactam, 12% to quinolones, 10% to third-generation cephalosporins, 7% to aminoglycosides and 7% to carbapenems [9, 14]. In recent years, some case reports have also described new isolates carrying the extended-spectrum β-lactamase gene [15, 16]. In our case, the antibiotic treatment regimen (cefixime → amoxicillin and potassium clavulanate → moxifloxacin and ganciclovir) was adjusted several times, although its clinical efficacy was poor. But the infection was effectively controlled after 6 days of treatment with meropenem. However, it is worth noting that there have been reports of new cases of infections resistant to carbapenems [17, 18].

*Raoultella ornithinolytica* also has the *hdc* gene that encodes for histidine decarboxylase, an enzyme with catalyzes the conversion of histidine to histamine. Therefore, infected patients may develop skin symptoms related to allergic reactions such as skin flushing, itching, and maculopapular formation [19, 20]. These clinical manifestations were confirmed in our case, and therefore in addition to appropriate antibiotics, antihistamine therapy was also necessary to successfully treat the patient.

In patients with these diseases it must also be noted that there may be some treatment limitations as follows. Firstly, because no pathogenic bacteria were found in body fluid cultures in our case, further drug susceptibility tests could not be carried out. Next-generation sequencing also failed to identify a clear drug-resistant gene locus and could not provide us with guidance for further adjustment of antibiotic therapy. Under the premise that the use of cephalosporins, penicillin, and quinolone antibiotics had not worked, we finally administered the carbapenem drug, meropenem to our patient. Secondly, before the next-generation sequencing results identified the pathogen we could only indirectly diagnose and provide empirical treatment based on the patient’s medical history, symptoms, signs, and the results of routine examinations. As a consequence, the allergic rash was mistakenly regarded as a rash caused by a viral infection and therefore ganciclovir was added to the treatment regime.

Although previous case reports have mainly suggested that *R. ornithinolytica* infections are more likely to occur in immunocompromised persons, our case indicates that the bacteria may also infect healthy people in the community. The clinical characteristics of the onset of infection in healthy individuals are not only infection-related symptoms, but also symptoms of allergic reactions to bacterial components, which are quite different from those reported previously for immunocompromised infections. We also found in our case, that although *R. ornithinolytica* did not have a clear drug resistance gene, the pathogenic bacteria were not sensitive to common clinical antibiotic treatments and administration of carbapenem drugs was required to treat the infection. As a novel clinical pathogen that is able to spread gradually, *R. ornithinolytica* should not be regarded as miscellaneous bacteria that can only contaminate tissue samples. Physicians should be aware of its risk to cause a community infection with high drug resistance leading to an increased risk of death from sepsis. Clinical manifestations should therefore be identified quickly and appropriate control measures initiated at an early stage. Broad-spectrum antibiotics combined with antihistamine treatment could be considered before accurate microbiological results are obtained.

## Data Availability

All data analysed during this study are included in this published article. Data sharing is not applicable to this article as no datasets were generated or analysed.

## Abbreviations

SLNE: Superficial lymph node enlargement
*R. ornithinolytica*: *Raoultella ornithinolytica*
HAIs: Healthcare-associated infections
